# Superelongation of Liquid Metal

**DOI:** 10.1002/advs.202105289

**Published:** 2022-02-07

**Authors:** Xiangpeng Li, Lu Cao, Bing Xiao, Fangxia Li, Junhui Yang, Jie Hu, Tim Cole, Yuxin Zhang, Mingkui Zhang, Jiahao Zheng, Shiwu Zhang, Weihua Li, Lining Sun, Xiaoqian Chen, Shi‐Yang Tang

**Affiliations:** ^1^ College of Mechanical and Electrical Engineering Soochow University Suzhou 215000 China; ^2^ National Innovation Institute of Defense Technology Beijing 100071 China; ^3^ School of Automation Northwestern Polytechnical University Xi'an 710072 China; ^4^ Department of Electronic, Electrical and Systems Engineering University of Birmingham Edgbaston Birmingham B15 2TT UK; ^5^ CAS Key Laboratory of Mechanical Behavior and Design of Materials Department of Precision Machinery and Precision Instrumentation University of Science and Technology of China Hefei 230026 China; ^6^ School of Mechanical Materials Mechatronic and Biomedical Engineering University of Wollongong Wollongong NSW 2522 Australia

**Keywords:** elongation, Galinstan, liquid metal, Marangoni instabilities, wearable sensors

## Abstract

The ability to control interfacial tension electrochemically is uniquely available for liquid metals (LMs), in particular gallium‐based LM alloys. This imparts them with excellent locomotion and deformation capabilities and enables diverse applications. However, electrochemical oxidation of LM is a highly dynamic process, which often induces Marangoni instabilities that make it almost impossible to elongate LM and manipulate its morphology directly and precisely on a 2D plane without the assistance of other patterning methods. To overcome these limitations, this study investigates the use of an LM–iron (Fe) particle mixture that is capable of suppressing instabilities during the electrochemical oxidation process, thereby allowing for superelongation of the LM core of the mixture to form a thin wire that is tens of times of its original length. More importantly, the elongated LM core can be manipulated freely on a 2D plane to form complex patterns. Eliminating Marangoni instabilities also allows for the effective spreading and filling of the LM–Fe mixture into molds with complex structures and small features. Harnessing these excellent abilities, a channel‐less patterning method for fabricating elastomeric wearable sensors is demonstrated to detect motions. This study shows the potential for developing functional and flexible structures of LM with superior performance.

## Graphical abstract

Tuning interfacial tension is an effective way to manipulate fluids at small scales. This study investigates the liquid metal–iron particle mixture that can suppress surface Marangoni instabilities, thereby allowing for superelongation of the liquid metal core electrically in an electrolyte to form a thin wire. The elongated core can be manipulated freely on a 2D plane to form complex patterns.

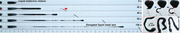

## 1 Introduction

For fluids at small length scales, surface forces tend to dominate in comparison to body forces. Therefore, tuning interfacial tension between the liquid–gas or liquid–liquid interface has been proven to be an effective way to manipulate fluids at small scales. For conventional aqueous and organic solutions, methods such as surface chemistry (e.g., using surfactant),^[^ ^1^, ^2^ ^]^ introducing nanoparticles at the interface,^[^ ^3^ ^]^ changing temperature,^[^ ^4^, ^5^ ^]^ and electrostatics^[^ ^6^ ^]^ have been explored to tune interfacial tension. These methods, however, are less effective for liquid metals (LMs), which exhibit the highest interfacial tension (>400 mN m^−1^) among all liquids.

Liquid metals are a special type of liquid that possess both metallic and fluidic properties at (or near) room temperature. Common metallic elements and alloys such as mercury (Hg, m.p. of −38.8 °C) and sodium–potassium (NaK, m.p. of −12.6 °C) are in the liquid state at or below room temperature. Nonetheless, mercury is notorious for its toxicity, while NaK is explosively reactive when contacted with water, thus reducing their usability in wider applications. Alternatively, gallium (Ga) has a melting point of 29.8 °C, which is close to room temperature; adding other metals (e.g., indium, tin, aluminum, zinc, silver) into Ga to form alloys can bring the melting point down to (or below) room temperature.^[^ ^7^ ^]^ Among which, eutectic Ga–indium (EGaIn; 75 wt% Ga and 25 wt% In; m.p. of 15.4 °C) and Ga–In–tin (Galinstan; 67 wt% Ga, 20.5 wt% In, and 12.5 wt% Sn; m.p. of ≈11 °C) are the two most commonly used liquid metal (LM) alloys with many superior properties.^[^ ^8^, ^9^, ^10^, ^11^ ^]^ The combination of both metallic and fluidic properties, together with their low toxicity and relatively good biocompatibility, means that Ga‐based LMs offer exciting opportunities for applications in electronics,^[^ ^12^, ^13^ ^]^ microfluidics,^[^ ^14^, ^15^ ^]^ energy,^[^ ^16^, ^17^ ^]^ chemistry,^[^ ^7^, ^18^ ^]^ and biomedical research.^[^ ^19^, ^20^, ^21^ ^]^

Surface chemistry introduced by ligands has shown its effectiveness in lowering the interfacial tension of Ga‐based LMs, which is evidenced by the production of smaller sized nanoparticles with the presence of surfactants when using sonication.^[^ ^22^, ^23^, ^24^ ^]^ Alternatively, electrical methods including electrocapillarity^[^ ^25^, ^26^, ^27^ ^]^ and electrowetting^[^ ^28^ ^]^ have been explored to moderately tune the interfacial tension of LM, and yet electrochemical reduction/oxidation to remove/grow an Ga oxide layer is so far regarded as the most effective way to tune the large tension from its maximum value (hundreds of mN m^−1^) all the way to near zero.^[^ ^29^, ^30^, ^31^ ^]^ Theoretically, the elimination of surface tension allows LMs to be elongated indefinitely. However, the electrochemical oxidation of LM is highly dynamic, often accompanied with the formation of fractal shapes for an oxidized LM droplet, and the fractals may eventually break away from the electrode due to Marangoni instabilities.^[^ ^32^, ^33^ ^]^ This makes it difficult to directly use the electrochemical method to precisely and stably manipulate the morphology of LM, particularly in an unconstrained 2D plane.^[^ ^34^ ^]^

Elongating LM droplets can form conductive wires or patterns with semicircular cross sections, enabling innovative applications in soft electronics and microfluidics.^[^ ^12^, ^35^ ^]^ Several methods have been developed for overcoming the large surface tension of LM for elongation, such as direct writing on a substrate,^[^ ^36^, ^37^ ^]^ filling patterned microchannels,^[^ ^38^, ^39^ ^]^ and magnetically stretching LM mixed with ferromagnetic particles.^[^ ^40^, ^41^, ^42^ ^]^ However, these methods often require complicated operations and expensive instruments (e.g., dedicated printers, photolithography facilities, and electromagnetic arrays with controllers), or has limited ability to form thin wires with large aspect ratios. Alternatively, droplets of LM (or its colloidal mixture with other metal particles) embedded in elastic substrates can be mechanically shaped upon stretching, and the elongated LM droplet fillers can provide enhanced thermal conductivity for the composites.^[^ ^43^, ^44^, ^45^ ^]^ And yet, this method requires an external polymer matrix to hold the shape of the elongated LM droplets.

To take the advantage of the simplicity of the electrochemical method for manipulating surface tension of LM, and meanwhile eliminating the instability problem associated with this method, here, we present that LM encapsulated in a LM–iron (Fe) particle (LM–Fe) shell can be readily guided and elongated by tens of times using electrochemistry to form stable thin wires, which has never been observed and achieved using bare LM. We investigated the mechanism behind this extraordinary elongating ability, and explored parameters affecting the elongating performance. Next, we demonstrated guiding and manipulating the elongated LM on a 2D plane without the constraint from a channel. In addition to elongation, we showed that the LM–Fe mixture possesses a significantly enhanced ability compared to bare LM to be electrochemically patterned into molds with complex shapes and small features. Finally, harnessing the extraordinary elongating ability of the LM–Fe mixture, we demonstrated a channel‐less method for embedding the lengthened LM wire within an elastomer for making wearable strain sensors.

## 2 Results and Discussion

**Figure 1:**
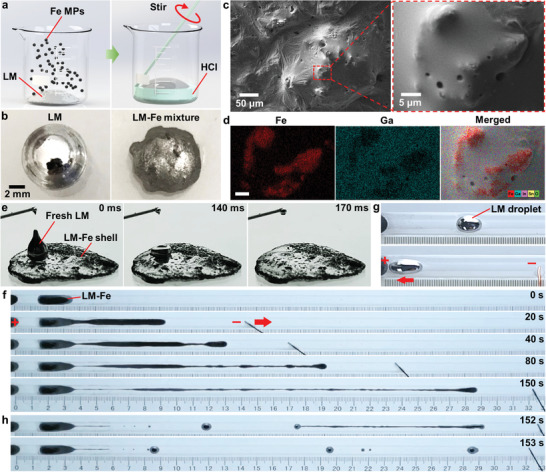
Preparation and elongation of the LM–Fe mixture. a) Schematic showing the process for making the LM–Fe mixture. b) Images showing the difference between bare Galinstan and the LM–Fe mixture in NaOH solution, the LM–Fe mixture has a rough surface and is gray colored. c) SEM images of the surface of the LM–Fe mixture. d) EDS element mappings for the magnified area given in (c). e) Snapshots showing the encapsulation of fresh LM into the LM–Fe shell. f) Snapshots showing the electrochemical elongation of the LM core within the LM–Fe mixture. g) Actuation of a bare Galinstan droplet toward the anode driven by the Marangoni force in NaOH solution. h) Breaking of the LM wire to form LM droplets.

The fabrication process for obtaining the LM–Fe mixture is illustrated in **Figure** **1**a. In brief, we mixed 200 µL Galinstan and Fe particles (diameter of ≈50 µm, contents ranging from 2 to 20 wt%) in a beaker with the presence of a hydrochloric acid (HCl) solution (8 wt%) for ≈15 min at room temperature. We next removed the HCl solution and thoroughly washed the mixture with deionized (DI) water. Compared to bare Galinstan, the surface of the LM–Fe mixture is no longer pristine and is covered by a thick layer of solid shell, as shown in Figure 1b. However, the inner core of the LM–Fe mixture remained to be liquid like bare Galinstan. The scanning electron microscope (SEM) image of the LM–Fe shell shows the rough surface of the mixture (Figure 1c), and the energy‐dispersive X‐ray spectroscopy (EDS) mappings for characteristic elements (Figure 1d) indicate that Fe particles are covered by Galinstan LM. The corresponding EDS spectrum is given in Figure S1 (Supporting Information). We further conducted X‐ray diffraction analysis (XRD) to identify the components within the LM–Fe shell, as shown in Figure S2 (Supporting Information). The spectrum shows that the shell mainly contains metallic Ga and *α*‐Fe, and we did not detect the formation of Ga–Fe alloy within the mixture. The liquid core of the LM–Fe mixture can be removed using a syringe and fresh Galinstan LM can rapidly merge within the shell, indicating that the LM–Fe shell is reusable, as shown in Figure 1e.

To demonstrate the extraordinary electrochemically induced elongation of the LM core encapsulated within the LM–Fe shell, we placed a droplet of the LM–Fe mixture (200 µL) into a poly(methyl methacrylate) (PMMA) channel (length of 300 mm, width of 4 mm, and height of 5 mm) filled with NaOH solution (0.5 m). A graphite electrode connected to the anode was fixed at the left end of the channel and a copper wire connected to the cathode was placed on the right side of the droplet. Upon the application of an 8 V DC voltage, the LM core came out of the shell and elongated toward the copper electrode. Moving the electrode toward the right end of the channel while carefully maintaining an ≈40 mm distance between the front end of the lengthened LM and the electrode can induce further elongation of the LM core, forming a long LM thin wire with a maximum length of ≈250 mm (≈12 times of its original body length), as shown in Figure 1f (see also Movie S1 in the Supporting Information). We define the body ratio (BR) as the ratio between the length of the elongated LM wire and the original length of the LM–Fe droplet in a channel. The minimum width of the LM wire is ≈300 µm. In contrast, a bare Galinstan droplet (200 µL) rapidly moved toward the anode due to the Marangoni force upon the application of an 8 V DC potential drop along the channel (Figure 1g), which is similar to previously reported results.^[^ ^46^, ^47^ ^]^ The wire eventually broke and detached from the LM–Fe shell at the thin region and beaded up to form droplets, indicating the importance of the LM–Fe tail for enabling the elongation, as shown in Figure 1h.

**Figure 2:**
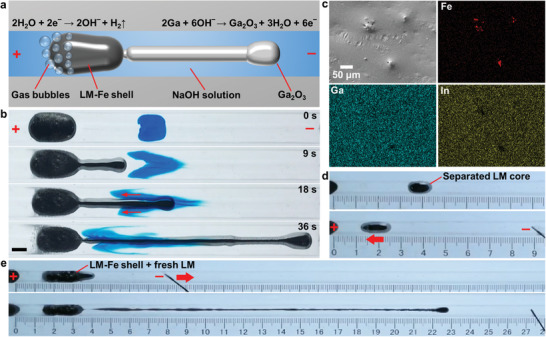
Investigation of the mechanism of elongation. a) Schematic showing the electrochemical reactions on the elongated LM–Fe mixture. b) Snapshots showing the generation of Marangoni flows along the elongated LM core. The LM–Fe mixture (400 µL) contains 10 wt% of Fe particles. Scale bar is 5 mm. c) SEM image and EDS element mappings of the surface of the separated LM core. d) Actuation of a droplet of the separated LM core (200 µL) in NaOH solution (0.5 mol L^−1^). e) Snapshots showing the electrochemical elongation of the LM–Fe mixture refilled with fresh Galinstan.

We believe the key factor for inducing such a large elongation of the LM–Fe mixture is due to the formation of a stable oxide on the elongated LM surface with the presence of the LM–Fe shell (tail), which can improve the deformability of the droplet by significantly reducing the surface tension.^32^ The LM–Fe mixture is bipolarized upon applying a DC potential, and **Figure** **2**a depicts the proposed electrochemical reaction on the cathodic and the anodic poles of the LM–Fe droplet when lengthened. We observed the formation of gas bubbles on the surface of the LM–Fe shell (cathodic pole) during elongation, which is due to the electrochemical reduction of protons to form hydrogen gas (H_2_O + 2e^−^ → 2OH^−^ + H_2_↑). On the other hand, the end of the elongated LM core (anodic pole) is oxidized (2Ga + 6OH^−^ → Ga_2_O_3_ + 3H_2_O + 6e^−^) so that the surface tension is diminished. The addition of Fe particles facilitates the electrochemical generation of hydrogen gas, thereby allowing for electron transfer to oxidize the LM core to induce the elongation. The unidirectional elongation is probably due to the existence of Marangoni forces, which the effective interfacial tension at the region closer to the copper electrode being lower than the region further away.^[^ ^32^, ^34^ ^]^ We verify this hypothesis by tracking the flow of the surrounding liquid along the elongated LM using food dye, as shown in Figure 2b (see also Movie S2 in the Supporting Information), in which a flow toward the anode was observed for the NaOH solution, indicating the generation of Marangoni flows. For bare LM droplets, nonuniform electrochemical growth of an oxide layer causes an inhomogeneous distribution of stress on the LM surface; such an inhomogeneities can then be amplified to cause perturbations to result in a finger‐like pattern.^[^ ^48^ ^]^ Without Fe particles, the NaOH solution can dissolve the oxide to make the finger‐like pattern unstable and eventually cause the elongated part to break away from the body. We believe that the incorporation of Fe particles enables the formation of a thick and stable oxide that can suppress fingering instabilities.

After elongation, the SEM image and EDS mappings of the separated LM core show that it contains a much lower amount of Fe particles, as evidenced by its relatively smooth surface with sparsely distributed Fe particles (Figure 2c). The corresponding EDS spectrum given in Figure S3 (Supporting Information) indicates that the LM core contains negligible amount of Fe. This makes the appearance and the actuating behavior of the LM core similar to bare Galinstan in NaOH solution, as shown in Figure 2d. We conducted further analysis of the Fe particle distribution by obtaining EDS mappings of the cross section of a frozen LM–Fe droplet, as detailed in Figure S4 (Supporting Information). The solid shell contains a much higher content of Fe particles, while the inner core remains to be liquid and contains almost no Fe particles. The Fe particle content becomes higher in areas closer to the surface. Interestingly, after refilling the LM–Fe shell with fresh Galinstan (see Figure 1e), the mixture droplet can be electrochemically lengthened again to form a LM wire, reaching a similar BR close to ≈12, as shown in Figure 2e. This indicates that the elongation process is highly repeatable as long as the LM–Fe shell exists.

**Figure 3:**
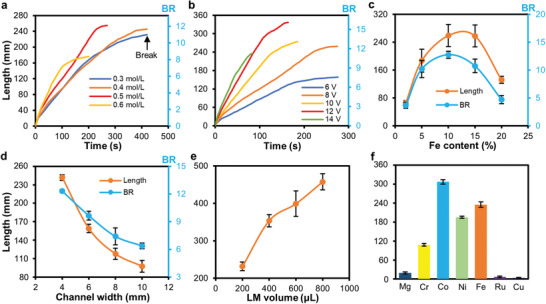
Investigating factors that affect the elongating performance. a) Plots of length and BR of the elongated LM wire versus time in NaOH solutions of different concentrations. b) Plots of length and BR versus time for LM–Fe droplets (200 µL, 10 wt% Fe content) elongated with different voltages. c) Plots of the maximum length and BR versus Fe particle contents for LM–Fe droplets elongated in a 0.5 mol L^−1^ NaOH solution. d) Plots of the maximum length and BR versus channel width for LM–Fe droplets (200 µL, 10 wt% Fe content) elongated in a 0.5 mol L^−1^ NaOH solution. e) Plots of the maximum length and BR versus volume of LM–Fe droplets (10 wt% Fe content, elongated in a 0.5 mol L^−1^ NaOH solution). f) Comparison of the maximum lengths of the elongated LM wires for LM mixed with different types of metal particles (10 wt%). The value of the error bar is the standard deviation of five measurements.

We further investigated and characterized factors that may affect the elongating performance of the LM–Fe mixture, including the NaOH concentration, the applied voltage, the widths of the PMMA channel, the content of Fe particles, the volume of the LM–Fe mixture, and the type of solid particles mixed with the LM. To understand the effect of the NaOH concentration, a droplet of LM–Fe mixture (200 µL, 10 wt% of Fe particles) was placed into a straight channel (width of 4 mm and depth of 5 mm) and NaOH solutions with different concentrations (from 0.3 to 0.6 mol L^−1^) were tested. We recorded the length of the elongated LM core with respect to time when an 8 V DC voltage was used for inducing elongation, as shown in **Figure** **3**a. In NaOH solution, the dissolution of oxide species competes with oxide deposition during electrochemical oxidation. We can see that the length of the LM wire increases almost linearly until break occurs, and the elongating speed becomes faster as the NaOH concentration increases. The higher speed might be due to the increased Marangoni forces induced along the lengthened LM wire at a higher concentration. Meanwhile, the largest elongating length increases for higher NaOH concentrations until reaching 0.5 mol L^−1^, beyond which the elongating length conversely reduces. This is because a high NaOH concentration can dissolve the surface oxide more rapidly to increase surface tension, which makes the thin wire easier to break. The optimum concentration of NaOH is ≈0.5 mol L^−1^.

We next explored the influence of the applied voltage on the elongating performance. In doing so, a droplet of LM–Fe mixture (200 µL, 10 wt% of Fe particles) was placed into a straight channel filled with 0.5 mol L^−1^ NaOH solution. We recorded the length of the elongated LM core with respect to time when different DC voltages were used for guiding the elongation, as shown in Figure 3b. We found that increasing the voltage can effectively lead to faster elongation and a larger wire length. However, break of the LM wire may occur earlier if the voltage is too large. Therefore, the optimum operating voltage is ≈12 V. The Fe particle content also significantly affect the elongating performance, as shown in Figure 3c. For a content less than 10 wt%, a higher elongating length can be achieved when increasing the particle concentration. Since Ga has a lower standard electrode potential (*E* ^o^ = −0.56 V) than that of Fe (*E* ^o^ = −0.44 V), it acts as the anode in a galvanic cell and loses electrons (oxidized) when submerged in an electrolyte. Therefore, increasing the content of Fe particles will accelerate the formation of Ga oxide, thereby efficiently lowering the surface tension for yielding better elongating performance.^31^ However, when the Fe particle content exceeds 15 wt%, a decrease in the elongating length was observed. This is probably because the elongated LM contains more Fe particles, which in turn weakens the thin region of the LM wire to make it easy to break. We used a content of 10 wt% of Fe particles for the rest of the experiments unless specified otherwise.

For droplets of the LM–Fe mixture with the same volume (200 µL), using channels of different widths also leads to different maximum elongating lengths, as shown in Figure 3d. The longest length (also the largest BR) was achieved using a channel with a narrow width of 4 mm, and increasing the channel width leads to compromised elongating performance. Moreover, a larger length of the LM wire can be achieved by increasing the volume of the droplet of the LM–Fe mixture, as shown in Figure 3e. In addition to Fe, we also tested LM mixtures containing other types of microparticles (10 wt%), including magnesium (Mg, *E* ^o^ = −2.36 V), chromium (Cr, *E* ^o^ = −0.90 V), cobalt (Co, *E* ^o^ = −0.28 V), nickel (Ni, *E* ^o^ = −0.26 V), ruthenium (Ru, *E* ^o^ = 0.25 V), and copper (Cu, *E* ^o^ = 0.52 V), as shown in Figure 3f. Interestingly, we found that metals with the *E* ^o^ large than that of Ga but smaller than 0 V provides good performance for inducing the elongation (see Figure S5 in the Supporting Information for an example comparting the elongating performance of LM–Mg, LM–Co, and LM–Cu droplets). This can be partially explained using reactions of galvanic cells that Ga can be oxidized in an electrolyte when connected to a metal with a higher *E* ^o^, however, it is still unknown why metals with a *E* ^o^ large than 0 V prevents the elongation. This phenomenon is subject to further investigation in our future study.

**Figure 4:**
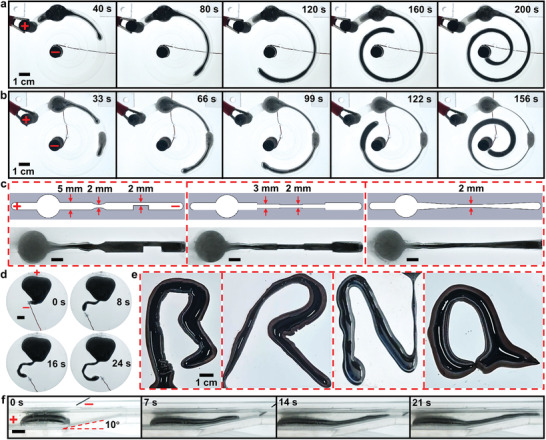
Further exploration of the ability for elongating LM. a) Elongating a droplet of the LM–Fe mixture along a spiral channel. b) Elongating two droplets of the LM–Fe mixture along a spiral channel for achieving a faster speed. c) Elongating the LM–Fe mixture along complex channels. Scale bars are 5 mm. d) Elongation and manipulation of the LM–Fe mixture on a 2D plane. Scale bar is 1 cm. e) Elongated LM core with complex shapes of “B,” “R,” “N,” and “a.” f) 3D elongation of the LM–Fe mixture along a slope. Scale bar is 5 mm.

Previous sections show the guided elongation of the LM–Fe mixture in a straight channel using a movable copper electrode. To further explore the ability of the elongation method, we designed a series of proof‐of‐concept experiments to demonstrate the superelongation in curved channels and channels with nonuniform cross sections using a pair of fixed electrodes. To show this, we placed a droplet of the LM–Fe mixture (1 mL) into a spiral channel (length of 290 mm, width of 10 mm, and height of 5 mm) filled with NaOH solution (0.5 mol L^−1^), and two graphite electrodes were fixed at the two ends of the channel. Upon the application of an 8 V DC voltage, the LM–Fe mixture smoothly elongated along the spiral channel until reaching the cathode, as shown in **Figure** **4**a (see also Movie S3, Part 1, in the Supporting Information). Interestingly, when two droplets of the LM–Fe mixture were placed apart in the spiral channel, once the lengthened LM core from the droplet closer to the anode touches the second droplet, the two LM cores were connected to enable a faster elongating speed to fill the spiral channel, as shown in Figure 4b (see also Movie S3, Part 2, in the Supporting Information).

To further explore the ability of the elongated LM to pass a slit or a thin channel, we designed three channels with complex shapes and varied width (the narrowest width of 2 mm), as shown in Figure 4c. Droplets of the LM–Fe mixture (800 µL) successfully lengthened along all channels without breaking upon applying a 6 V DC voltage between the electrodes, representing the flexibly of the elongated LM with the ability to adapt to the surrounding environment (Figure 4c; see also Movie S4 in the Supporting Information).

In addition to elongating in confined channels, we demonstrated the ability of the elongation and manipulation of the LM–Fe mixture in an open 2D space. In doing so, a droplet of LM–Fe mixture (2 mL, 10 wt% of Fe particles) was placed in a shallow dish filled with NaOH solution (0.5 mol L^−1^), a copper foil was fixed on the inner wall of the dish to act as the anode, and a movable copper wire was connected to the cathode for elongating the LM core. Figure 4d shows that the direction of elongation can be readily controlled freely using the copper electrode (voltage of 6 V) on a 2D plane, and the LM core formed a letter “C” on the dish. Harnessing the unique ability, we accomplished writing more complex letters with sharper angles using the elongated LM core, including “B,” “R,” “N,” and “a,” as shown in Figure 4e (see also Movie S5 in the Supporting Information). Finally, we also examined the ability of elongating the LM–Fe mixture in a 3D space, as shown in Figure 4f, in which we demonstrated the elongation of the LM core (1.5 mL) along a slope with an angle of inclination of 10° upon the application of an 8 V DC voltage (see also Movie S6 in the Supporting Information).

**Figure 5:**
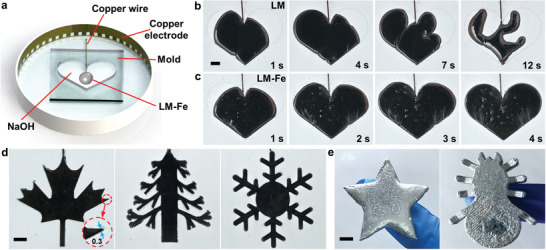
Improved spreading performance of the LM–Fe mixture in molds. a) Schematic of the experimental setup for the spreading of bare LM and the LM–Fe mixture. Electrochemical spreading of b) a bare Galinstan LM droplet and c) a droplet of the LM–Fe mixture into a heart‐shaped mold. d) Electrochemical spreading droplets of the LM–Fe mixture in molds of various complex shapes. The smallest feature of the mold is ≈300 µm. e) Electrochemistry‐assisted shape casting of the LM–Fe mixture. Scale bars are 5 mm.

In addition to elongation, we found that the LM–Fe mixture possesses other unique properties that are superior to bare LM, including electrochemically induced spreading. To show this, we placed a droplet of LM–Fe mixture or bare Galinstan in a PMMA mold filled with NaOH solution (0.5 mol L^−1^) and connected the droplet to the anode, while a circular Cu electrode was used to surround the mold and connected to the cathode, as shown in **Figure** **5**a. Figure 5b shows the electrochemical oxidation and spreading process of a bare Galinstan droplet (1 mL) in the mold as a result of the near‐zero effective interfacial tension. However, the Marangoni instabilities due to gradients in effective interfacial tension makes the spreading process unreliable, and eventually causes fragmentation (see also Movie S7, Part 1, in the Supporting Information).^[^ ^32^ ^]^ In contrast, uniform and rapid spreading of LM without fragmentation was observed for the LM–Fe mixture (1 mL), replicating the heart‐shaped pattern of the mold, as shown in Figure 5c (see also Movie S7, Part 2, in the Supporting Information). To further present the capability of this unique property, we achieved the spreading of the LM–Fe mixture (1 mL) in molds of various shapes with small and complex features, such as maple leaf (smallest feature of ≈300 µm), pine tree, and snowflake, as shown in Figure 5d (see also Movie S8 in the Supporting Information). After spreading, lowing the temperature can solidify the LM–Fe mixture filled in molds to cast components with complex shapes, as shown in Figure 5e.

**Figure 6:**
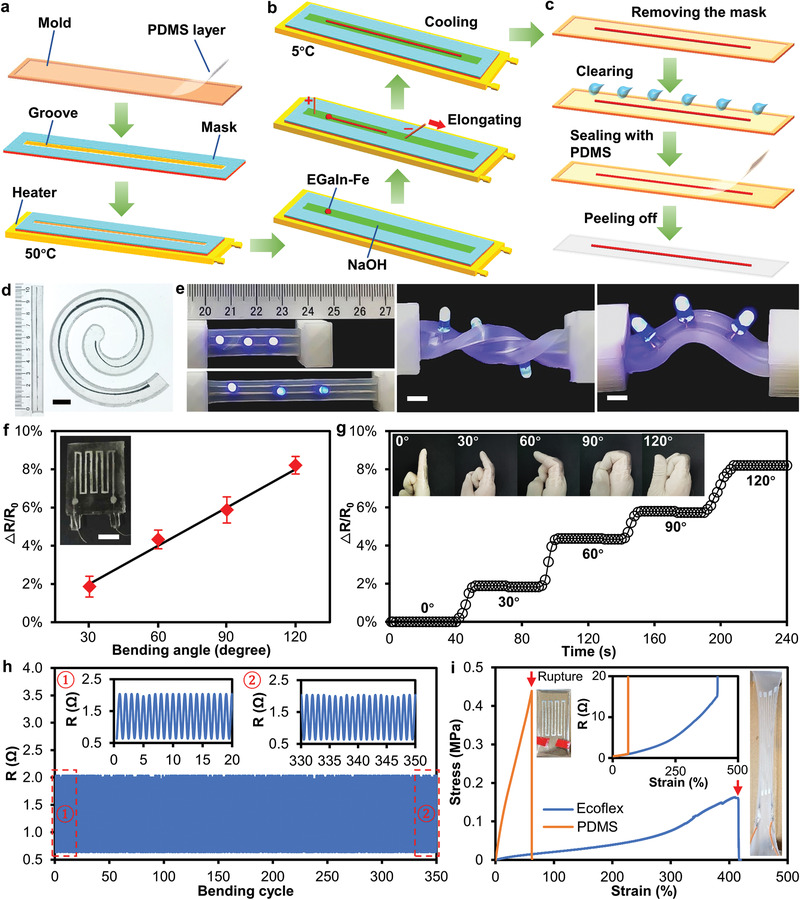
Channel‐less patterning method enabled by the electrochemical elongation of LM. a‐c) Schematics showing the process flow for the channel‐less patterning of LM wires. d) Patterned straight and spiral LM wires in PDMS. Scale bar is 1 cm. e) LED array assembled on the Ecoflex encapsulated LM conductive paths under different deformations. Scale bars are 5 mm. f) Relative resistance change of the wearable sensor under different bending angles. The inset shows the actual image of the sensor. Scale bar is 1 cm. The value of the error bar is the standard deviation of three measurements. g) Change of relative resistance recorded at different bending angles. The inset shows the corresponding images of the bended finger at different angles. h) Resistance‐time curve of the strain sensor during a cyclic bending experiment. i) Strain–stress curves for strain sensors encapsulated in PDMS or Ecoflex. The inset images show the stretched sensors right before rupture. The middle inset shows the change of resistance of the sensors upon stretching.

Unlike conventional patterning methods that need to inject LM into microfluidic channels, the formation of LM wires induced by electrochemical elongation allows for the channel‐less patterning of LM to form conducting paths without the need for the soft lithography process. We used EGaIn instead of Galinstan to make the mixture (EGaIn–Fe) in this experiment since solidification of the elongated LM wire is needed during the fabrication process. Such a patterning method is illustrated in **Figure** **6**, in which we can specify the process in the following major steps:

1. Spin‐coat a thin layer (thickness of 500 µm) of polydimethylsiloxane (PDMS) on the 3D printed polylactide (PLA) substrate and cure at 80 °C for 2 h. Then, attach a rectangular mask with a straight open‐top groove (length of 150 mm, width of 10 mm, and height of 2 mm) to the PDMS layer and heat the substrate to 50 °C using a heater (Figure 6a).
2. Add 1 mL NaOH solution (0.5 mol L^−1^) and a droplet of EGaIn–Fe mixture (400 µL) into the groove. Elongate the EGaIn–Fe mixture along the groove using a 10 V DC voltage until the desired length is reached. Then, turn off the heater and inject a cold NaOH solution (5 °C) to freeze the LM wire before removing the voltage (Figure 6b).
3. Remove the mask and clean the solution and impurities around the LM wire using DI water. Finally, use an additional layer of PDMS (thickness of 500 µm) to encapsulate the LM wire and then remove the sandwiched structure from the substrate (Figure 6c).

Taking the advantage of the versatility of the LM elongation method, we achieved straight and spiral‐shaped stretchable LM wires encapsulated in PDMS, as shown in Figure 6d. Figure S6 shows straight wires with different lengths of 100, 150, and 200 mm. The above‐described procedures are universal, which can also be applied to make sensors encapsulated in more stretchable elastomers (such as Ecoflex and ethylene vinyl acetate) to further enhance the stretchability.^[^ ^49^, ^50^ ^]^ Figure 6e shows that LM conductors embedded in Ecoflex exhibit excellent flexibility and stretchability. No cracks or compromise in conduction was observed when straining (85%), twisting (by 180°), and bending (by 60°) the conductor, which is of great importance for making wearable sensors.

For showing a proof‐of‐concept application, a wearable strain sensor for monitoring the movement of a finger was fabricated using electrochemically elongated LM wires embedded in PDMS elastomer. The sensor (width of 30 mm and length of 34 mm) consisted of a serpentine LM line resistor (line width of 1 mm and length of 20 mm for each loop) and two Cu wires (500 µm in diameter) inserted to connect the two terminals of the LM wires, as shown in the inset of Figure 6f. The sensor exhibits an excellent linear relationship between the bending angle and the change of resistance (Figure 6f), and it can be attached to a human forefinger for motion analysis. We measured the relative resistance change of the sensor as the finger joint bent at different angles (0∼120° with 30° increment), as shown in Figure 6g, in which we can see the measurements remained stable at different angles. This set of experiments shows the feasibility of the channel‐less patterning method for making useful devices.

We attached the sensor to a robotic hand to perform long‐term cyclic bending experiments for ≈350 cycles to verify its stability and robustness (see Figure S7 for the experimental setup). In each cycle (120° bending angle, 2 s period), the resistance of the sensor linearly increased from ≈0.63 to ≈2.03 Ω, and then returned to 0.63 Ω at the same speed. Figure 6h shows the resistance‐time curve of the strain sensor. The two insets respectively show the resistance curves of the first 20 and the last 20 cycles. The change of resistance of the sensor was basically stable, with a resistance change of less than 0.2% throughout the experiment.

We further performed tensile experiments on sensors encapsulated by PDMS or Ecoflex to compare their sensing performance and tensile limits, as shown in Figure 6i. From the strain–stress curves obtained for these sensors, we can see that Ecoflex has a much lower modulus and offers a greater tensile limit (≈420%, see the right inset of Figure 6i) than that of PDMS (≈64%, see the left inset). The middle inset of Figure 5i shows the change of resistance of the two sensors during the tensile tests, in which we can see that both sensors have the similar linear response to a small strain (<200%). However, the Ecoflex encapsulated sensor has a much larger working range due to its high tensile limit.

## 3 Conclusion

In summary, we presented the unprecedented electrochemically induced superelongation of the liquid metal–iron particle mixture that can reach tens of times of its original body length, whereas locomotion is induced for bare LM instead of elongation under the same conditions. The LM core in the mixture can be elongated along channels with complex structures or manipulated freely on a 2D plane with the guidance of an electrode. The elongated LM can also climb a slope, showing its potential to induce 3D manipulation. We characterized the properties of the LM–Fe mixture and investigated the mechanism behind the elongation. We found that the LM–Fe shell formed on the surface plays the key role in forming a stable but dynamic oxide layer for diminishing surface tension and meanwhile stabilizing the elongated LM wire. In addition, unlike bare LM, for which the spreading induced by electrochemical oxidation is unstable due to the Marangoni instability, the LM–Fe mixture can rapidly and effectively spread in molds with complex structures and small features upon electrochemical oxidation. Harnessing these excellent abilities, we demonstrated a channel‐less patterning method for fabricating stretchable LM conductors embedded in an elastomeric matrix. We further demonstrated the use of the patterned LM conductor for making strain sensors to detect motions of human fingers. Owing to the unique but extraordinary elongating ability of the LM–Fe mixture, the reported phenomenon has the potential to be further explored for fabricating innovative functional and flexible structures of LM with superior performance.

## 4 Experimental Section

### Materials and Instrument

Galinstan alloy (67% Ga, 20.5% In, and 12.5% Sn) was prepared by dissolving In and Sn metals in Ga. All materials have a purity of 99.99%. The iron particles with a diameter from 45 to 50 µm were purchased from Shanghai Yu‐sui Welding Material. SYLGARD 184 Silicone Elastomer Curing Agent and SYLGARD 184 Silicone Elastomer Base were purchased from Dow Corning, American. Ecoflex 00‐30 was purchased from Bentley Advanced Materials, UK. Graphite electrodes were purchased from Donguan Tangxia Xiangyang Graphite Co. Ltd. HCl and NaOH solutions used in this study were freshly made before all experiments. All channels used in experiments were fabricated by milling transparent PMMA plates. DC voltages were provided by a DC power supply (IT6432, ITECH, China). The robotic hand used for the cyclic bending test was sponsored by the Suzhou JODELL Robotics Co. LTD (JQ3‐5). Tensile tests were performed using a material testing machine (YK‐Y0026, Yaoke Equipment), and the change of resistance was monitors using a multimeter (Fluke 8845A, Fluke UK).

### Videos and Photos

Videos were captured using a DSLR camera (5D MARK2, Canon, Japan), and the snapshots were extracted from these videos. The velocity data was obtained using a high‐speed camera (HERO 5, GoPro, USA). SEM images and EDS element mappings were taken using a Merlin Compact SEM (Zeiss, German). The X‐ray diffraction (XRD) data was obtained using an X‐ray diffraction system (D8 Discover Plus, Bruker, Germany) at ambient temperature.

## Conflict of Interest

The authors declare no conflict of interest.

## Supporting information

Supporting Information

Supplemental Movie 1

Supplemental Movie 2

Supplemental Movie 3

Supplemental Movie 4

Supplemental Movie 5

Supplemental Movie 6

Supplemental Movie 7

Supplemental Movie 8

## Data Availability

The data that support the findings of this study are available from the corresponding author upon reasonable request.
