# Supplementary material for: Superelongation of Liquid Metal
Source: Adv Sci (Weinh). 2022 Feb 7;9(11):2105289. doi: 10.1002/advs.202105289 (PMC9008437; doi:10.1002/advs.202105289)
Supplement: Supplementary file 1 — Supporting Information [file ADVS-9-2105289-s008.pdf]

ADVANCED SCIENCE
Open Access

Supporting Information

for *Adv. Sci.*, DOI: 10.1002/advs.202105289

Super-Elongation of Liquid Metal

Xiangpeng Li, Bing Xiao, Lu Cao, Fangxia Li, Junhui Yang, Jie Hu, Tim Cole, Yuxin Zhang, Mingkui Zhang, Jiahao Zheng, Shiwu Zhang, Weihua Li, Lining Sun, Xiaoqian Chen*, Shi-Yang Tang**

Supporting Information

Super-Elongation of Liquid Metal

Xiangpeng Li, Bing Xiao, Lu Cao, Fangxia Li, Junhui Yang, Jie Hu, Tim Cole, Yuxin Zhang, Mingkui Zhang, Jiahao Zheng, Shiwu Zhang, Weihua Li, Lining Sun, Xiaoqian Chen*, Shi-Yang Tang**

X. Li, B. Xiao, and L. Cao contributed equally to this work

Prof. X. Li; F. Li; J. Yang; J. Hu; M. Zhang; Prof. L. Sun
College of Mechanical and Electrical Engineering, Soochow University, Suzhou 215000, China

Prof. B. Xiao
School of Automation, Northwestern Polytechnical University; Xi'an, 710072, China.

Dr. L. Cao, Prof. X. Chen
National Innovation Institute of Defense Technology; Beijing, 100071, China.
Email: chenxiaoqian@nudt.edu.cn

T. Cole, J. Zheng, Y. Zhang, Dr. S.-Y. Tang
Department of Electronic, Electrical and Systems Engineering, University of Birmingham, Edgbaston, Birmingham, B15 2TT, UK.
Email: S.Tang@bham.ac.uk

Prof. S. Zhang
CAS Key Laboratory of Mechanical Behavior and Design of Materials, Department of Precision Machinery and Precision Instrumentation, University of Science and Technology of China, Hefei, 230026, China
Email: swzhang@ustc.edu.cn

Prof. W. Li.
School of Mechanical, Materials, Mechatronic and Biomedical Engineering, University of Wollongong, Wollongong, NSW 2522, Australia

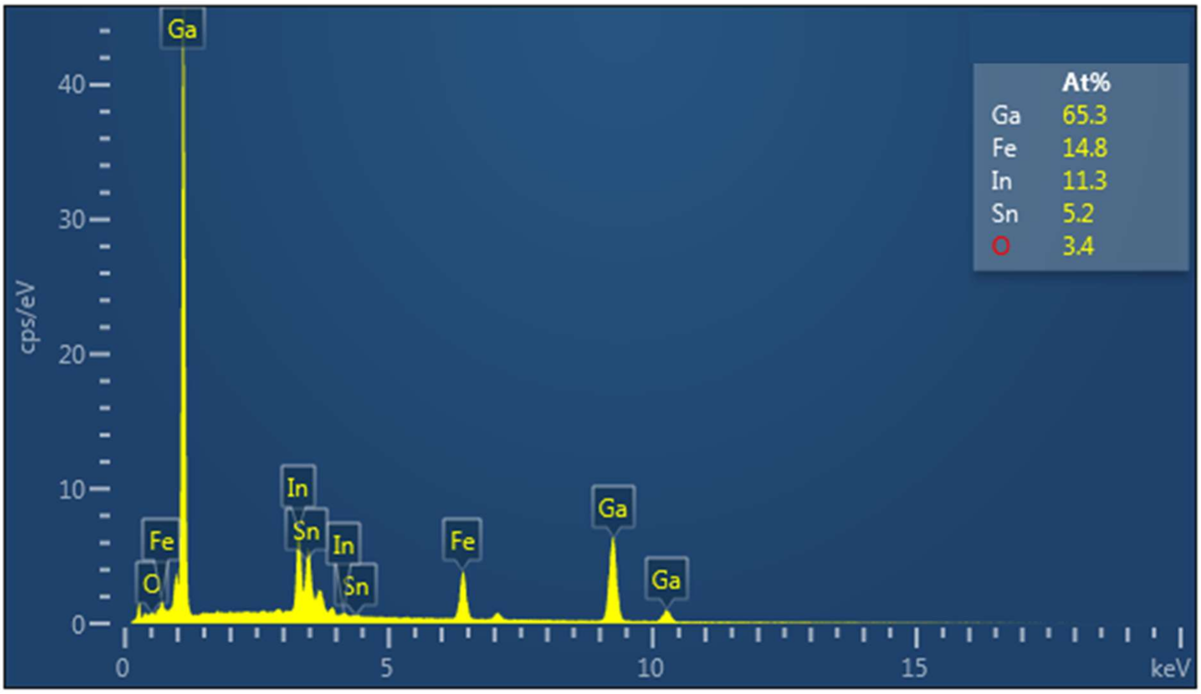

Figure S1. EDS spectrum for the LM-Fe shell of the mixture.

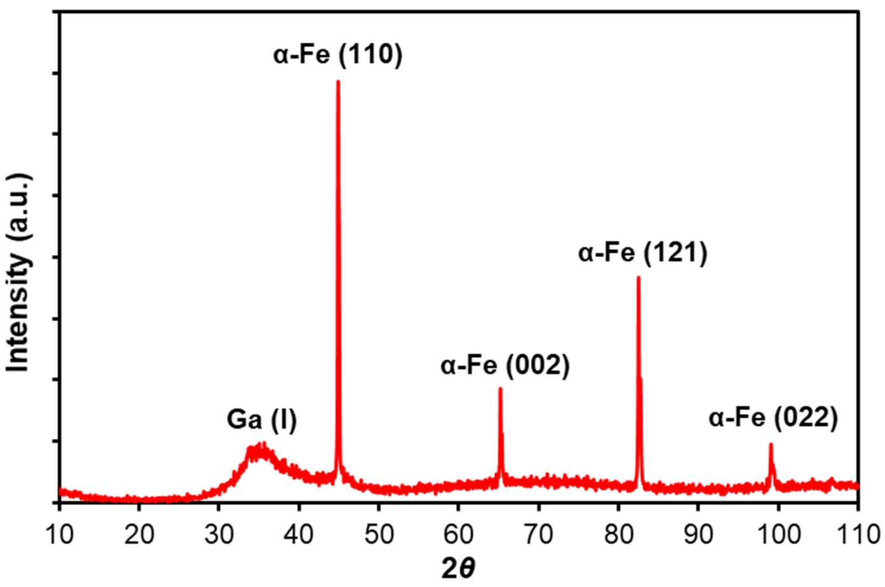

Figure S2. XRD spectrum for the LM-Fe shell of the mixture.

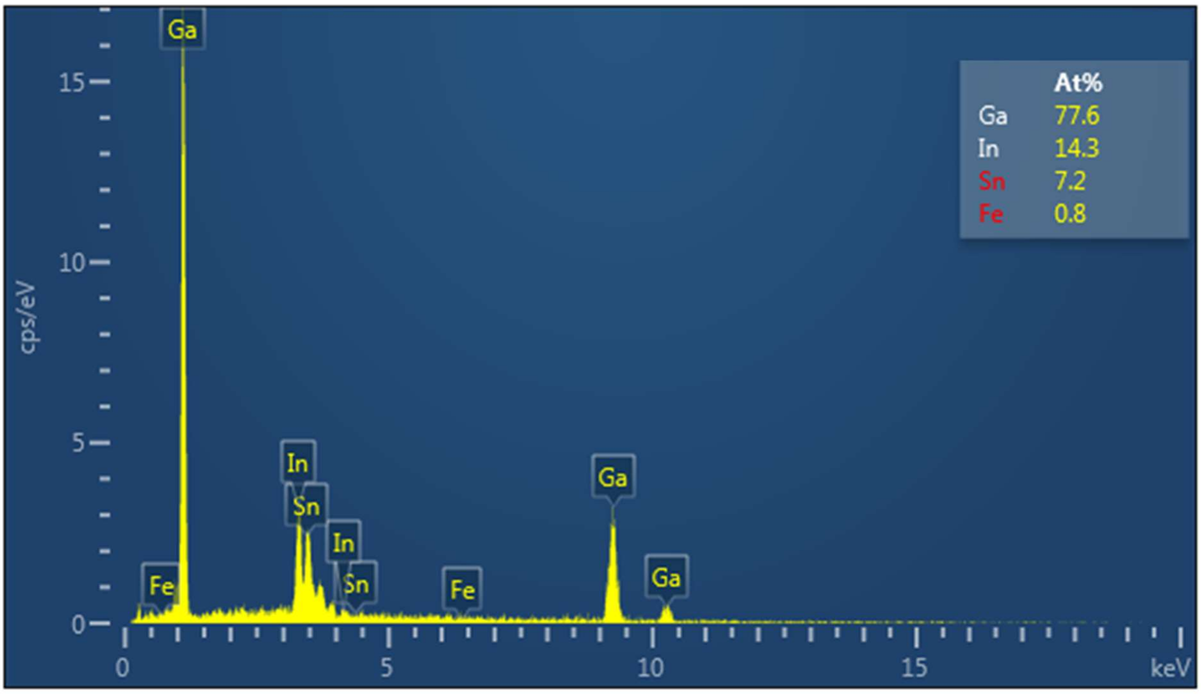

Figure S3. EDS spectrum for the separated LM core from the LM-Fe mixture.

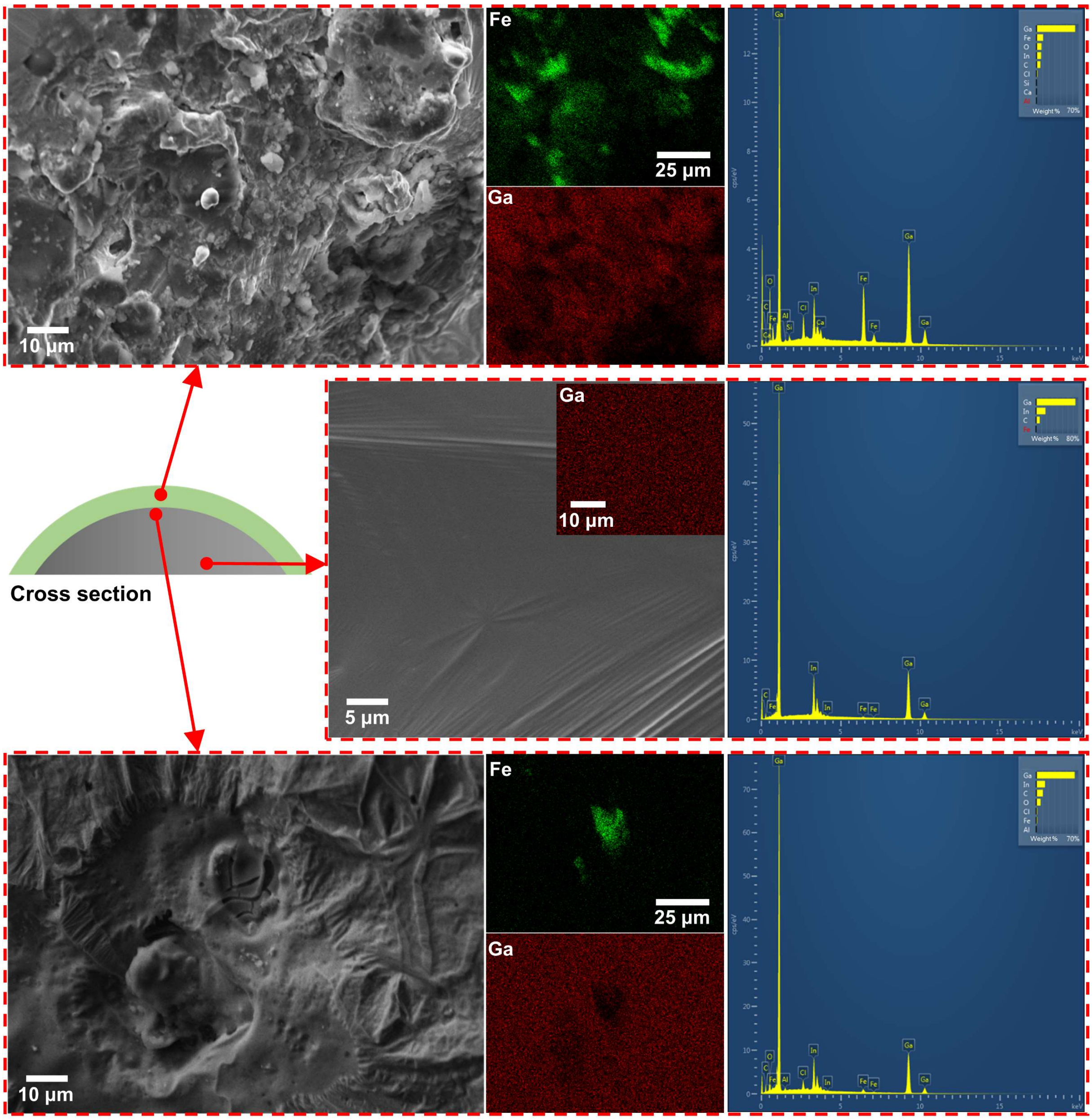

Figure S4. SEM images, EDS mappings (Ga and Fe), and EDS spectra for the cross section of a droplet of the LM-Fe mixture. To give a better illustration of the LM-Fe shell structure, we froze and cut a droplet of the LM-Fe mixture and conducted SEM and EDS analysis for the cross section. It is clear that the solid shell contains a much higher content of Fe particles, while the inner core remains to be liquid and contains almost no Fe particles. The Fe particle content becomes higher in areas closer to the surface.

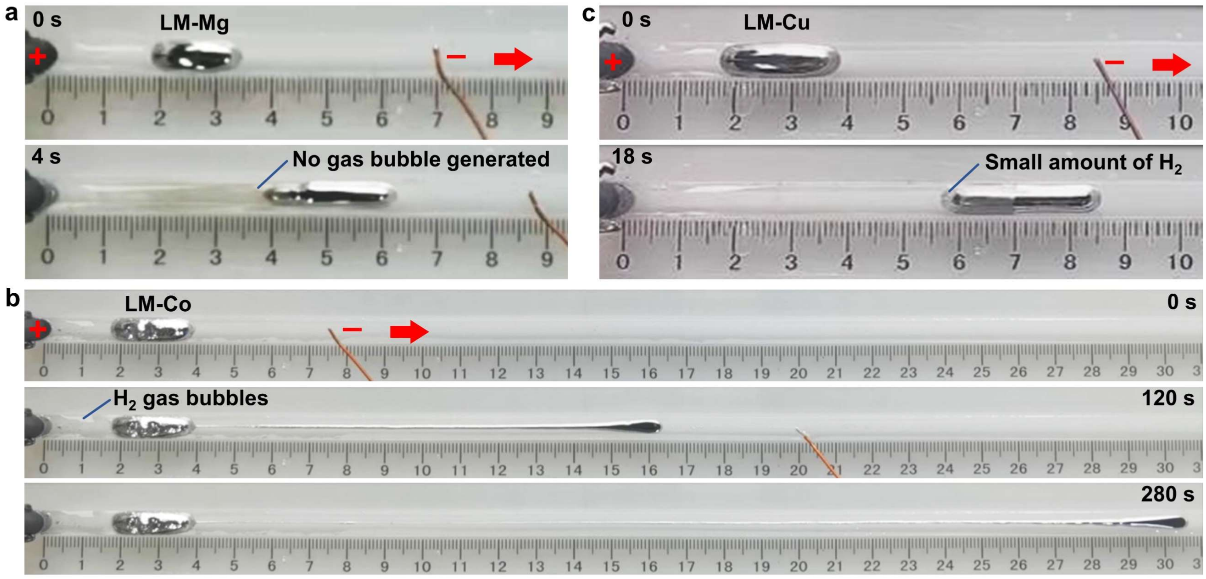

Figure S5. Comparison of the elongating performance between (a) LM-Mg, (b) LM-Co, and (c) LM-Cu droplets. Unlike Mg which has a low standard electrode potential (−2.36 V), the high standard electrode potential of Co (−0.28 V) induces the oxidation of LM, thereby facilitating the elongation of the droplet. However, the elongating performance is compromised for the case of Cu, which has a positive standard electrode potential of 0.52 V.

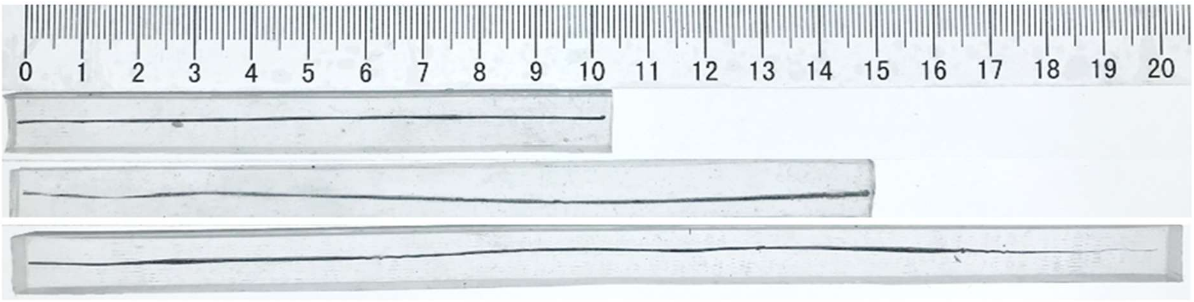

Figure S6. Elongated LM wires of different lengths encapsulated in PDMS.

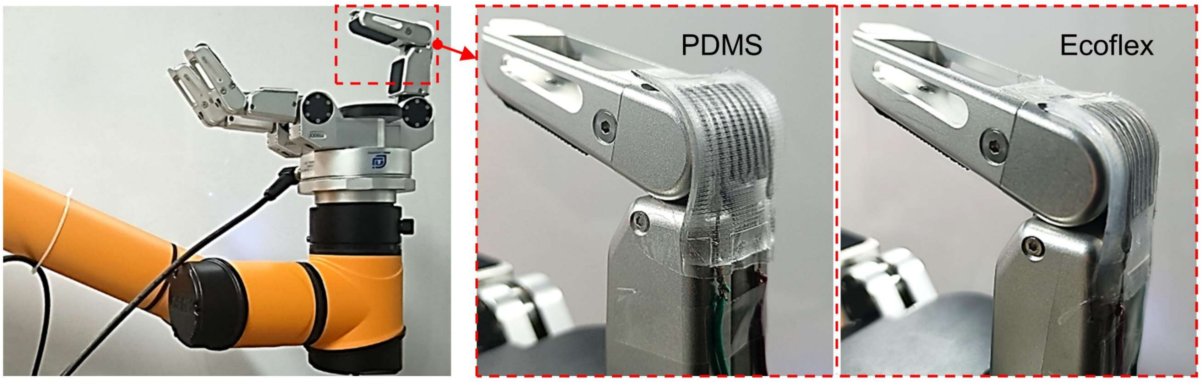

Figure S7. Experimental setup for the cyclic bending test of the strain sensor. The strain sensor was attached to a finger of the robotic hand for collecting data with controlled bending angle (120º) and cycle period (2 s).

Movie S1: Elongation of the LM-Fe mixture in a channel

Movie S2: Induction of Marangoni flows along the LM wire

Movie S3: Elongation of the LM-Fe mixture in a spiral channel

Movie S4: Elongation of the LM-Fe mixture through thin channels

Movie S5: Manipulation of the LM-Fe mixture on a 2D plane

Movie S6: Elongation of the LM-Fe mixture along a slope

Movie S7: Spreading of Galinstan and the LM-Fe mixture in a mold

Movie S8: Spreading of the LM-Fe mixture in complex molds
